# Exuberant case of erythema elevatum diutinum in a patient infected with HIV and hepatitis B virus^☆^^☆☆^

**DOI:** 10.1016/j.abd.2019.02.013

**Published:** 2020-02-12

**Authors:** Sayuri Aparecida Hirayama, Cezar Arthur Tavares Pinheiro, Isabelle Maffei Guarenti, Danise Senna Oliveira

**Affiliations:** a Department of General Medicine, Teaching Hospital, Universidade Federal de Pelotas, Pelotas, RS, Brazil; b HIV/AIDS Specialized Care Service, Universidade Federal de Pelotas, Pelotas, RS, Brazil; c Empresa Brasileira de Serviços Hospitalares, Teaching Hospital, Universidade Federal de Pelotas, Pelotas, RS, Brazil

**Keywords:** Cutaneous, Hepatitis B virus, HIV, Leukocytoclastic, Vasculitis

## Abstract

Erythema elevatum diutinum is a small vessel vasculitis which is benign, rare, and chronic. It is clinically characterized by violaceous, brown, or yellowish plaques, nodules, and papules. It has been associated with autoimmune, infectious, and neoplastic processes. The following case describes a patient with hepatitis B virus and human immunodeficiency virus with CD4 count < 200 mm^3^, HIV-seropositive for 16 years, and diagnosed with hepatitis B virus at the hospital. The patient was treated with oral dapsone 100 mg/day, showing regression after seven months of treatment. The authors found three cases in the literature of association of erythema elevatum diutinum, human immunodeficiency virus, and hepatitis B virus.

## Introduction

Erythema elevatum diutinum (EED) is a distinct form of cutaneous leukocytoclastic vasculitis, first recorded in 1878 by Hutchinson and later in 1879 by Bury. It was officially named by Radcliff-Crocker and Williams in 1892^1^ (apud Jose SK, 2016, p. 81). It occurs predominantly in adults from 40 to 60 years, being slightly more prevalent in men.^2^ Although its pathogenesis is still unknown, it is believed that immune complexes are deposited on the wall of venules and other vessels by continuous antigen stimulation or other infections. Therefore, EED is associated with hematological, autoimmune, neoplastic, and infectious diseases, such as human immunodeficiency virus (HIV) infection and hepatitis. This deposition of immune complexes leads to activation of the complement cascade *via* IL-8, with neutrophil chemotaxis, releasing lysozymes, collagenases, myeloperoxidases, and hydrolases that induce fibrin deposition and cholesterol crystals in the capillaries and venules, leading to damage.^1^, ^2^

This condition is characterized by papules, plaques, and nodules on the extensor surfaces of extremities, with a predilection for hands, feet, elbows, knees, and Achilles tendons, while sometimes being observed on the face and ears. These lesions occur symmetrically and bilaterally; they are initially soft and erythematous or purpuric, with occasional ulceration. Over time, lesions may develop with hypo- or hyperchromia in the event of regression. The nodular form is rarer, usually occurring in patients with HIV infection. Pruritus and burning pain in the lesions may be observed, as well as arthralgias and ocular alterations, such as nodular scleritis, panuveitis, autoimmune keratolysis, and peripheral keratitis.^1^

The authors describe the case of a patient infected with HIV and hepatitis B virus (HBV) presenting with leukocytoclastic vasculitis diagnosed by biopsy of skin lesions.

## Case report

**Figure 1:**
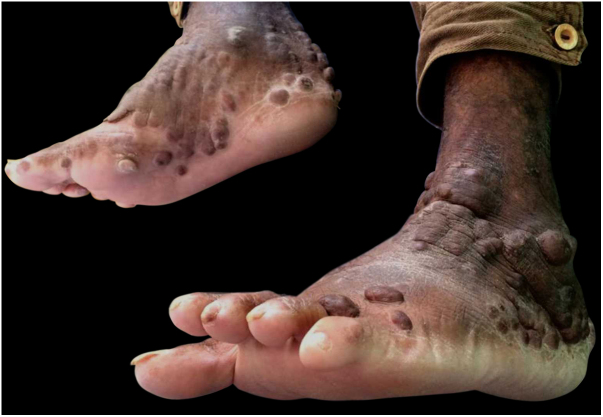
Medial aspect of right foot and lateral aspect of left foot with erythematous-brown plaques.

**Figure 2:**
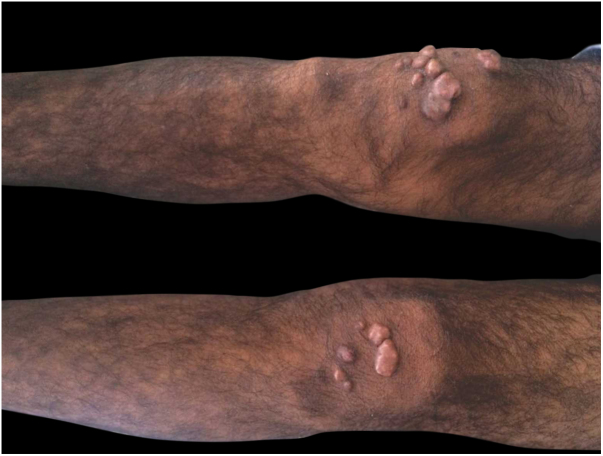
Brownish nodular lesions on the knees.

**Figure 3:**
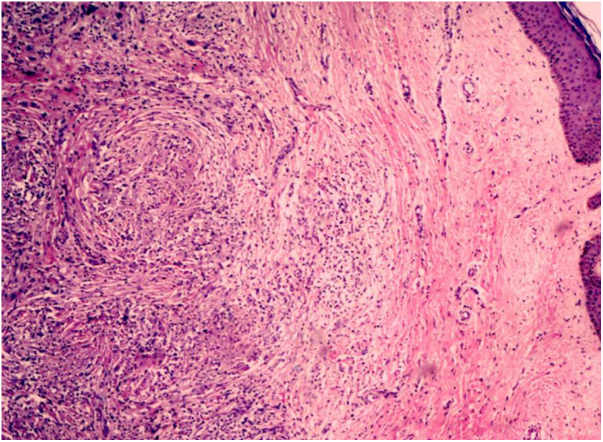
Anatomopathological examination of the skin biopsy demonstrating neutrophilic dermatitis, with marked leukocytoclasia and presence of fibrotic nodules surrounding the neutrophilic infiltrate, compatible with leukocytoclastic vasculitis (Hematoxylin & eosin, ×40).

**Figure 4:**
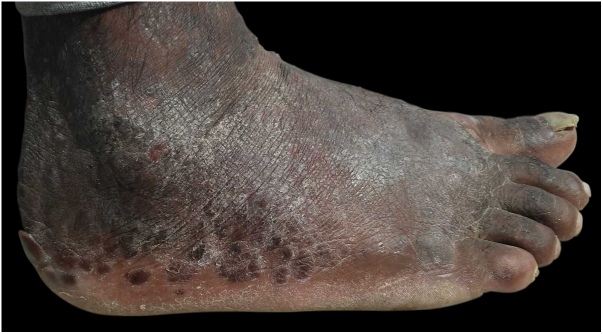
Lateral aspect of right foot after seven months of treatment, showing lesions regression.

This was a 43-year-old black male patient with a 16-year history of HIV infection who was using lamivudine + tenofovir + lopinavir/ritonavir (viral load 25,000 copies/mL and CD4 count of 39 cells/mm^3^). Three years before, a single and nodular lesion had appeared in the right calcaneus; others lesions on the extensor face of the left lower limb and left elbow were observed in subsequent months. All lesions were itchy and progressed in number and size over time. Physical examination disclosed symmetrically distributed erythematous-xanthochromic nodules on the knees and elbows, and linear plaques and erythematous-violaceous nodules on the ankles, toes, and plantar region (Figure 1, Figure 2). The patient denied visual alterations and arthralgias. In this hospitalization, the patient was diagnosed with neurotoxoplasmosis, pneumocystosis, and hepatitis B (HbsAg, total anti HBC, and HBEAg reagents with AST/TGO = 20 U/L and ALT/TGP = 11 U/L). Due to suspicion of tuberous xanthoma, a lipid profile was requested, which was normal. Kaposi's sarcoma was also suspected. Biopsies of two of the lesions were performed. The anatomopathological examination demonstrated neutrophilic dermatitis with marked leukocytoclasia and presence of fibrotic nodules surrounding the neutrophilic infiltrate (Fig. 3). Clinical and pathological correlation indicated EED. Treatment with dapsone 100 mg/day was initiated, which resolved the lesions within seven months (Fig. 4).

## Discussion

The diagnosis of EED is clinical and histopathological; in the early stages, the latter presents leukocytoclastic vasculitis with polymorphonuclear cells, macrophages, and histiocytes in the dermis, and in the late stage, granulation tissue, fibrosis, vascular proliferation, lymphohistiocytic inflammatory infiltrate, and focal areas containing neutrophils with leukocytoclasia. In the late stage, intra- and extracellular lipids (cholesterol deposits), although rare, may be observed. The differential diagnosis in the early phase may be Sweet's syndrome, pyoderma gangrenosum, facial granuloma, drug-induced rash, erythema multiforme, cutaneous porphyria, and bullous pemphigoid. In the late stage, the differential diagnosis considers dermatofibroma, fibromatosis, necrobiotic xanthogranuloma, and tuberous xanthoma. In patients with HIV infection, Kaposi's sarcoma and bacillary angiomatosis should also be considered.^2^

The first choice of treatment is dapsone.^3^ Alternatives are colchicine, tetracyclines, niacinamide, and systemic corticosteroids such as prednisolone.^4^ Topical betamethasone may also be used. New therapies with 5% topical dapsone and plasmapheresis have been described.^3^ In the late stage of the disease, there is little response to dapsone, given the predominant fibrosis. In this case, intralesional corticosteroids or lesion excision are the treatments of choice^5^ The disease has a prolonged duration, with reports of spontaneous resolution ranging from five to ten years.^3^ Relapse may occur after dapsone discontinuation.

In the literature, approximately 25 cases of EED and HIV infection have been described,^6^ one of which in Brazil,^7^ and three cases of HIV/HBV/EED co-occurrence.^8^ EED is most commonly seen in patients with a CD4 count < 200 cells/mm^3^, and both immunosuppression and antigen-antibody reactions caused by HIV and HBV are believed to be the triggering factors of this disease. Nonetheless, in the study by Muratori et al.,^9^ in four out of five patients with HIV infection, the triggering factor was streptococcal infection. In these patients, the nodular form is the most prevalent,^10^ and the palmoplantar region may be involved, as in the present case. Differential diagnosis should include bacillary angiomatosis, Kaposi's sarcoma, and rheumatoid nodules.^1^

The present case is relevant, due to the few reports of EED/HIV/HBV patients in the literature. It is common to find skin lesions in immunosuppressed patients, which may suggest diseases of various etiologies. Thus, histopathological confirmation is essential to establish the diagnosis, the stage of the disease, and guide treatment.

## Financial support

None declared.

## Authors’ contributions

Sayuri Aparecida Hirayama: Approval of the final version of the manuscript; conception and planning of the study; elaboration and writing of the manuscript; obtaining, analyzing, and interpreting the data; critical review of the literature; critical review of the manuscript.

Cezar Arthur Tavares Pinheiro: Preparation and writing of the manuscript; effective participation in research orientation; intellectual participation in propaedeutic and/or therapeutic conduct of studied cases; critical review of the manuscript.

Isabelle Maffei Guarenti: Approval of the final version of the manuscript; effective participation in research orientation; critical review of the manuscript.

Danise Senna Oliveira: Intellectual participation in propaedeutic and/or therapeutic conduct of studied cases.

## Conflicts of interest

None declared.
